# Curiosity across the adult lifespan: Age-related differences in state and trait curiosity

**DOI:** 10.1371/journal.pone.0320600

**Published:** 2025-05-07

**Authors:** Mary C. Whatley, Kou Murayama, Michiko Sakaki, Alan D. Castel

**Affiliations:** 1 Department of Psychology, University of California, Los Angeles,; 2 Department of Psychology, Western Carolina University, Cullowhee, North Carolina, United States; 3 Hector Research Institute of Education Sciences and Psychology, University of Tübingen, Tübingen, Germany; 4 Research Institute, Kochi University of Technology, Kochi, Japan; Nanyang Technological University, SINGAPORE

## Abstract

Maintaining curiosity in older age may be a key predictor of successful aging, but prior research on the relationship between curiosity and age is mixed, with mounting evidence showing that curiosity declines with age. However, there is evidence suggesting that state curiosity – a situational feeling of curiosity in response to information – may increase with age. Prior work has largely not adequately differentiated state and trait curiosity when examining its relationship with age. In a large lifespan sample (pilot study *N* = 193; preregistered main study *N* = 1,218), we assess trait curiosity and state curiosity (using a trivia rating task) to examine the relationship between each construct and age. The results show that, in line with prior work, trait curiosity shows a negative relationship with age, but state curiosity shows a positive relationship with age, while controlling for demographic variables. The results suggest that curiosity may have a more complex relationship with age than previously considered, which can have implications for engagement in cognitive activities in everyday life.

## Introduction

Curiosity is a construct that has been pondered and studied by psychologists and philosophers for centuries. It is a motivating influence that drives us to participate in hobbies, pursue education, and travel to experience new things. Curiosity is broadly defined as a desire to learn, experience, or explore new information or environments [see 1]. Trait curiosity, defined as people’s stable tendency to actively seek knowledge and information, has been shown to be correlated with a variety of positive traits in everyday settings. For example, in educational settings, curiosity is related to rates of student question asking [2] and academic performance [3]. Additionally, medical students with higher levels of curiosity report having deeper motives for studying and engaging in deeper study strategies when learning new information [4]. Beyond educational settings, curiosity is a predictor of job performance and learning at work [5]. In addition, those who show higher levels of trait curiosity also report more meaningful moments in their life [6]. Thus, curiosity can motivate learning and goal pursuit in a variety of settings.

As with many psychological constructs, curiosity can be measured as both a trait (i.e., a more general and stable feature) and a state (i.e., a momentary experience). Trait curiosity is typically captured by self-reported questions asking people about their general tendencies, and tangential constructs are often used as a proxy for curiosity (e.g., openness to experience, novelty seeking, love of learning). Trait curiosity is largely considered to be a positive personality trait that involves the tendency to find many things interesting and a motivation to explore novelty to satisfy personal interest [see 7]. While measurements differ, trait curiosity is considered to be a fairly broad form of general interest in many subjects. On the other hand, state curiosity typically reflects momentary motivation to gain information *in response to specific* learning materials. State curiosity is often (but not always) assessed with behavioral measures (e.g., question asking, information seeking) [3,8,9] and has been defined as a metacognitive state [10,11] and a drive to resolve a specific knowledge gap [8]. Thus, state curiosity arises in response to specific information and can be resolved when the gap is closed. Researchers have largely failed to distinguish between state and trait curiosity, viewing trait curiosity as simply the culmination of many instances of state curiosity [12, but see 13 for an exception]. People who frequently experience state curiosity may likely be more trait curious overall. However, there may be many instances in which people’s general tendency (i.e., trait curiosity) can be overridden by a situational trigger of curiosity (i.e., state curiosity). In the present research, we aim to examine both trait curiosity, assessed by questionnaires, and state curiosity, assessed in response to trivia questions, in the same individuals to separate contributions of these different forms of curiosity to our understanding of age-related differences.

## Age-related changes in state and trait curiosity

Different forms of curiosity may be especially important to maintain in older age [see 14]. Curiosity is often a primary motivator for older adults’ engagement in formal learning [15,16], with older adults reporting interest in learning as a reason for taking classes later in life more than for any utility reason (e.g., to learn a needed skill for work). Further, engagement in these types of stimulating cognitive activities has shown to protect against some age-related declines in cognitive abilities [17]. In one study, older adults who were more curious at a baseline measurement were shown to have a greater survival rate over a five-year period than those who were less curious [18]. These benefits led Sakaki et al. [14] to argue in a review of neuroscience and psychological research on curiosity that curiosity may be a key predictor of successful aging.

Although it has been established that maintaining trait curiosity throughout the lifespan can be beneficial for a variety of outcomes [5,6,14], the evidence regarding the relationship between chronological age and trait curiosity is somewhat mixed. Some evidence suggests that older age groups have higher trait curiosity, openness, or novelty seeking than younger age groups [19,20], though in some cases, these patterns show increases followed by declines or lack of sufficient data into older age (i.e., over 65 years of age) [21]. In line with this evidence, a comprehensive meta-analysis demonstrated that many personality traits become more stable across the lifespan [22], which may explain a plateau in trait curiosity in older age. Some evidence, however, suggests that trait curiosity may decline with age [23,24], drawing on support from socioemotional selectivity theory [SST; 25], a domain-general theory describing motivational shifts in terms of emotional and social goals. SST proposes that aging is associated with a limited future time perspective, which leads older adults to prioritize activities that satisfy goals that enhance their emotional experiences in the present, including emotional well-being and building close relationships. Younger adults, on the other hand, may aim to expand their social relationships and prioritize knowledge acquisition, which can satisfy long-term goals. These shifts in goals can influence the way we interact with others [26,27], learn and remember [28], and make decisions [29]. Based on the ideas posited by SST, knowledge acquisition goals should generally decline with age, as maintaining curiosity to learn in later life may not be an adaptive use of declining resources. Some studies have indeed shown that epistemic curiosity is lower in older adults [30,31], and that this is potentially driven by a more limited future time perspective [23] or emotion regulation strategies [24]. Additionally, while longitudinal evidence is limited with respect to curiosity, some evidence suggests that information seeking declines over time, though this may differ for men and women [32]. Taken together, the evidence and theoretical perspectives generally suggest that curiosity may decline as we age, although empirical evidence is somewhat mixed. Together with the pilot empirical study (described later), we hypothesized that trait curiosity would show a negative relationship with chronological age.

On the other hand, some work has shown higher levels of *state* curiosity in older adults compared to younger adults [8,33–35]. Specifically, older adults have reported greater curiosity to learn about specific information, such as trivia and assistive technology, and to explore new environments. Although the relationship between age and state curiosity is not well-established in the literature, together with the pilot study we conducted (described later), we hypothesized that, while people have decreased trait curiosity as they grow older, state curiosity, especially that triggered by materials that require semantic knowledge, would *increase* with age. As people age, they generally develop rich and wide semantic knowledge [36]. Existing knowledge has been acknowledged as an important source of curiosity [11,37–40]. Consequently, once people are exposed to concrete learning materials, older adults may be able to find more connections between the learning materials and their knowledge, resulting in increased curiosity to know the unlearned information [41].

The idea that older adults may be curious about specific information, like trivia questions that draw upon semantic knowledge, is also supported by Hess’ [42] selective engagement hypothesis (SEH), which argues that older adults perceive the cognitive costs (e.g., effort, fatigue) of engaging in a task as higher than younger adults because of declining resources and, as a result, are more selective about the tasks for which they choose to use limited cognitive resources. According to the SEH, not only are costs perceived as higher, but the benefits of engaging in a task become more salient as we age, and older adults may be less willing to engage in difficult cognitive tasks unless the self-relevant benefits are great enough. Because older adults are better able to learn information that is consistent with semantic knowledge [43–45], as well as if they have some expertise in a specific domain [46], older adults may experience less difficulty learning new information that expands on their prior knowledge (i.e., requiring fewer cognitive resources), and, therefore, may experience greater curiosity and intrinsic drive to learn this information than less relevant information that does not expand on prior knowledge. A recent study provided empirical evidence for this idea, showing in an information search task that older adults, in comparison to younger adults, showed a greater preference to deepen knowledge about a specific topic, rather than explore more topics [47].

To gain insights into the relationship between age and both state and trait curiosity, we conducted a pilot study (*N* = 193), in which we assessed state curiosity (i.e., curiosity to learn answers to trivia questions) and trait curiosity (assessed by three preexisting survey instruments). We observed a positive correlation between age and state curiosity (*r* =.16, *p* =.03), whereas age was negatively related to trait curiosity (*r* = -.29, *p* <.001). In the current preregistered study, we sought to confirm the findings that the relationship between age and curiosity would differ for measures of trait curiosity compared to state curiosity. A lifespan sample of adults (*N* = 1,218) completed a measure of trait curiosity and a trivia task that required them to rate their curiosity to learn the answers to trivia questions, which served as a measure of state curiosity. Given the prior work [24,30,31] and pilot results, we predicted trait curiosity would show a negative relationship with age. However, we also predicted that age would be *positively* associated with state curiosity in response to specific trivia questions, exhibiting an interesting contrast between trait and state curiosity with respect to aging. In other words, older adults have a general tendency to avoid new information (decreased trait curiosity), but once they are exposed to concrete learning materials, they are more engaged (e.g., increased state curiosity). Several studies have already suggested that older adults are more curious to learn specific information related to a task with small sample sizes [33–35] but we aimed to examine the robustness of these preliminary findings with a large sample size and a large number of questions to assess state curiosity.

### Method

We report how we determined our sample size and describe all data exclusions, manipulations, and all measures in the study. All data and analysis code are available at https://osf.io/6st5e/ [48], and the materials can be found online at https://motivationsciencelab.com/resources/. This study’s design, analysis plan, and hypotheses were pre-registered on the Open Science Framework (OSF).

### Participants

An *a priori* power analysis was conducted based on the effect size of the correlation between age and state curiosity in Fastrich et al. [49]. We conducted a power analysis using G*Power [50] with.95 power and alpha of.05 to detect a small effect size of *r* = 0.10 in a correlational model. The power analysis revealed that we needed a sample size of 1,289 participants. Based on our pilot study, we estimated that 25% of participants would be excluded according to our preregistered criteria to ensure data quality (see below). As such, we over-sampled; we planned and collected data from 2,000 participants from Amazon’s Mechanical Turk (MTurk) between March 2022 and August 2022.

### Task and measures

Participants’ data were excluded if they (1) reported looking up the answers to most of the questions, as this would affect curiosity ratings (*n* = 69), (2) were determined to be bots based on their responses to open-ended questions (*n* = 147), (3) had significant missing data from skipping most of the questions (*n* = 12) or from completing only one part of the study (e.g., completing the survey portion but not the trivia portion; *n* = 123), or (4) reported having significant problems with the task or their internet that affected their data (*n* = 156). In addition to these preregistered criteria, we also excluded participants who (5) were discovered to be a duplicate (*n* = 82), (6) reported having completed a task with the same questions before (*n* = 53), or (7) their reported birthday was more than one year off from their reported age (*n* = 140), indicating they lied about their age (an important variable in the current study). After the exclusion, the final sample of participants consisted of 1,218 adults aged 20–84 years (*M* = 44.4, *SD* = 15.5). Participant demographics are displayed in Table 1. All participants resided in the United States and were compensated $7.25 per hour for their participation.

**Table 1:** Participant Demographics.

	N	% of total	Mean (SD)
**Age**	1,213	99.6%	44.4 (15.5)
Not Reported	5	0.4%	--
**Education**	1,212	99.5%	16 years (2.11)
Not Reported	6	0.5%	--
**Household Income**	1,167	95.8%	$60,656 ($30,375)
Not Reported	51	4.2%	--
**Gender**			
Men	621	51.0%	--
Women	582	47.8%	--
Other	3	0.2%	--
Not Reported	12	1.0%	--
**Race/Ethnicity**			
American Indian/Alaska Native	10	0.8%	--
Asian/Pacific Islander	32	2.6%	--
Black/African American	94	7.7%	--
Hispanic or Latino/a/x	26	2.1%	--
White	1040	85.4%	--
More than one race	7	0.6%	--
Not reported	9	0.7%	--

To assess state curiosity, trivia questions were taken from a database normed by Fastrich et al. [49], in which questions had an average correct guess rate of approximately 16%, so that most participants would not know the answers to the questions. Trivia questions assessed many domains of general knowledge, including history, geography, animals, science, and food. Some examples were, “what is added to white sugar to make brown sugar?” (answer: molasses) and “what is the name of the biggest constellation in the sky?” (answer: hydra). To ensure the generalizability of the findings, all 244 trivia question-answer pairs from the database were used in the task, with a random 63 items selected for each participant. Each of the trivia questions was presented on the screen for 20 s along with a text box in a random order. Participants were told that they could make a guess, but if they did not think of a guess within 20 s, the page would automatically advance. Participants rated their curiosity to learn the answer to the question on a 1 (*not at all curious*) to 10 (*very curious*) scale, and then rated their confidence that they knew the correct answer on a 1 (*not at all confident*) to 10 (*very confident*) scale (both judgments self-paced). This state curiosity measure has been used in most of the experimental studies on curiosity using trivia questions and has shown relations with theoretically predicted variables (behaviors, other self-reported measures, neural activations; e.g., [13,51–53]). Participants were then shown the answer to the question for 2 s, and this process repeated for all 63 items. Each trial was separated by a page saying “the task will continue in a couple of seconds” which lasted 2 s. We included a trial at the halfway point that indicated to participants they were about halfway through the task and could take a short break to drink water, use the restroom, etc. if needed. Participants’ memory for the answers was not tested, as we were interested in curiosity ratings. On average, the trivia portion of the task took participants 34.87 minutes to complete.

To assess trait curiosity, we used the Epistemic Curiosity Scale [ECS; 54,55], which contains 10 items rated on a 1 (*almost never*) to 4 (*always*) scale. Items are statements such as, “I enjoy exploring new ideas” and “I spend hours on a problem because I can’t rest without the answer.” All items are averaged to create a total score. Chronbach’s alpha indicated good reliability (α =.82) in the current study.

In addition to trivia questions and ECS, we preregistered the intellectual curiosity (IC) facet from the openness to experience subscale of the larger Five Factor Inventory [56,57] as the other trait curiosity measure. However, this three-item scale showed very low reliability (α =.15) in our study, and thus we omitted the scale from analyses. The study also included survey items for boredom proneness, using the 8-item Boredom Proneness Scale [BPS; 58], scam susceptibility [59], and subjective age. These scales were not part of our primary research question, so we do not report the results here. Finally, participants also completed an open-ended question: “What do you think are the THREE most important words in life? Please list them below in any order.” This question allowed us to check for bots completing the task by assuring that responses were not random phrases and sounded human (see exclusion criteria above).

### Procedure

All procedures were approved by the University of California, Los Angeles institutional review board. Participants provided informed consent by checking a box online. Participants first answered demographic questions, including reporting their age, gender, race, education, income level, English fluency, and state of residence. Then half of the participants completed the survey portion of the task first, and the other half completed the trivia task first. For the trivia task, participants were first told that they would be studying trivia questions and answering questions about them, and they proceeded to the task after general instructions. For the survey portion of the task, participants simply responded to survey questions including ECS.

All participants reported their birthdate at the end of the entire task in order to match their birthday with their age. Participants were told that if they were not comfortable providing their exact birthday, they could report the correct month and year but change the day, as it was important to ensure that their age was accurate.

### Analysis plan

In all analyses, items that participants guessed correctly were filtered out of the data, as there is some evidence [11] that participants are less curious toward information they already know, and items already known to participants are qualitatively different from those unknown to participants. However, we analyzed the number of items participants correctly guessed and found that age was negatively related to number of items guessed correctly (*r* = -.12, *p* <.001), showing older adults correctly guessed fewer answers than younger adults. Participants who guessed more answers correctly reported higher trait (*r* =.10, *p* <.001) and state curiosity (*r* =.07, *p* =.021). Neither trait curiosity scores, *t*(1216) = 1.54, *p* =.123, nor state curiosity ratings, *t*(1216) = 0.96, *p* =.335, significantly differed by order (i.e., completing the survey questions or the trivia task first).

State curiosity scores were computed by averaging curiosity ratings across the 63 trivia question items (reliability =.988, based on generalizability theory). We also computed average confidence scores in a similar manner. In line with our preregistered analysis plan, we first conducted correlations between all variables (see Table 2) before following up with regression models. For trait curiosity, we conducted a multiple linear regression model predicting trait curiosity from age (centered) with gender (dummy coded, anchored on males), race (dummy coded, anchored on White), education (centered), and income (centered) as covariates. While we are not aware of empirical studies examining the relationship between these demographic variables and curiosity (except for gender; see [32]), these are common controlling variables used in aging research to estimate primary effects more accurately. The covariates described here were included to control for potential confounds and better estimate the effect of age. All included covariates were preregistered.

**Table 2:** Correlations between Trait Curiosity, State Curiosity, and Demographic Variables.

	Age	Education	Income	State Curiosity	Average Confidence	Trait Curiosity
Age	--					
Education	-.11***	--				
Income	-.04	.29***	--			
State Curiosity	.16***	-.06*	.03	--		
Average Confidence	-.33***	.32***	.09*	.12***	--	
Trait Curiosity	-.18***	.10***	.06*	.23***	.28***	--

For state curiosity, we first conducted a single-level multiple linear regression predicting state curiosity from age with gender, race, education, and income as covariates. Factor variables were dummy coded and continuous variables were mean centered. Next, to take into account the fact that participants saw different trivia questions, we conducted a mixed effects linear regression model using the lme4 package in R [60], with trial-level state curiosity ratings as the outcome variable and age, gender, race, education, and income as well as average confidence level as predictors. We included average confidence rating to control for a participant’s general tendency to provide high or low ratings on Likert scales. We included random intercepts of participants and items, as well as random slopes of items for the age variable. Donnellan, Usami, and Murayama [61] showed that this “random item slope regression” analysis ensures that the results are generalizable to the population of items and prevents the potential inflation of Type-1 errors.

In all analyses, we report the results regarding covariates in our models for completeness, although we do not generally interpret these effects, as our primary effect of interest was the age variable. There is evidence that interpreting secondary effects from a single regression model where there is a primary effect of interest is a fallacy [62]. Specifically, Westreich and Greenland show that estimates from covariates can be confounded, even though the estimate for the primary variable is not. Therefore, we do not interpret the coefficients of covariates. In addition, we did not recruit in such a way as to have comparable sample sizes across these variables. Therefore, the distribution of the covariates is uneven, making interpretation difficult.

## Results

**Fig 1:**
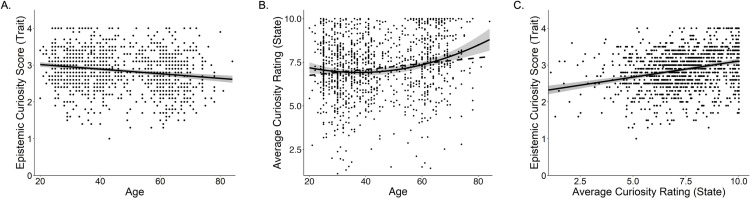
Relationships between Age, Trait Curiosity, and State Curiosity. Panel A shows the relationship between chronological age and scores on the Epistemic Curiosity Scale (ECS; trait curiosity). Panel B shows the relationship between chronological age and average curiosity ratings in the trivia paradigm (state curiosity), with the dashed line representing the linear relationship and the solid line representing the quadratic relationship. Panel C shows the relationship between average curiosity ratings from the trivia task and ECS scores.

The correlation matrix of the primary variables (state curiosity, confidence, and trait curiosity) as well as demographic variables is reported in Table 2. One notable observation is that state curiosity and trait curiosity were positively correlated, *r* =.23, *t*(1216) = 8.37, *p* <.01 (Fig 1C), indicating that those who have higher trait curiosity are likely to feel higher state curiosity when exposed to trivia questions.

### Age and trait curiosity

Consistent with our hypotheses, age and trait curiosity were *negatively* correlated, *r* = -.18, *t*(1211) = 6.54, *p* <.001 (Fig 1A). To further examine the robustness of this association, we conducted a linear regression analysis described in the analysis plan. The model revealed that age was a significant negative predictor of trait curiosity, *b* = -.006, *SE* = 0.001, *t*(1139) = 6.12, *p* <.001, Cohen’s *f*^2^ = 0.032. Looking at covariates, trait curiosity was also positively predicted by education, *b* = 0.02, *SE* = 0.008, *t*(1139) = 2.68, *p* =.007, Cohen’s *f*^2^ = 0.005. Neither gender, race, nor income were significant predictors of trait curiosity score (all *p*’s >.05).

Additionally, after examining the relationship visually, we conducted an exploratory analysis, which was not preregistered, to assess whether there was a quadratic relationship between age and trait curiosity. We found no significant quadratic relationship between age and trait curiosity, *b* < 0.001, *SE* = 0.00, *t*(1138), *p* =.62.

### Age and state curiosity

Consistent with our expectation, age and state curiosity were *positively* correlated (*r* =.16, *t*(1211) = 5.72, *p* <.001; Fig 1B – dashed line). To further examine the robustness of this association, we first ran a single-level regression described in the analysis plan, which revealed that age was a significant positive predictor of state curiosity, *b* = 0.02, *SE* = 0.003, *t*(1139) = 5.16, *p* <.001, Cohen’s *f*^2^ =.022. For the covariates, females also reported higher state curiosity than males, *b* = 0.21, *SE* = 0.10, *t*(1139) = 2.16, *p* =.031, Cohen’s *d* = 0.128. No other covariates significantly predicted state curiosity.

We next conducted our mixed effects model described in the analysis plan. The results showed that age was still a significant *positive* predictor of state curiosity, *b* =.02, *SE* = 0.003, *t*(1143) = 7.25, *p* <.001. Intraclass correlation coefficient (ICC) is a standard measure of effect size for a random slope effect [see 63]. The ICC for the age effect was 0.022. With regard to covariates, average confidence rating, *b* =.16, *SE* = 0.023, *t*(1142) = 7.01, *p* <.001, also significantly positively predicted state curiosity, while education, *b* = -0.09 *SE* =.025, *t*(1139) = 3.82, *p* <.001, had a significant negative relationship with state curiosity. Females were also significantly more curious than males, *b* = 0.24, *SE* = 0.10, *t*(1142) = 2.47, *p* =.014. Some racial differences emerged, such that African American participants were more curious than White participants, *b* = 0.55, *SE* = 0.18, *t*(1140) = 3.08, *p* =.002, and participants of more than one race were more curious than White participants, *b* = 1.74, *SE* = 0.617, *t*(1135) = 2.82, *p* =.005. No other predictors of s*t*ate curiosity were significant.

Finally, we added a quadratic age predictor to our model to test for the presence of a nonlinear relationship between age and state curiosity. This analysis was exploratory, as it was not preregistered and was conducted after examining the data visually. The model showed that there was a significant quadratic effect of age, *b* = 0.10, *SE* = 0.02, *t*(1141) = 3.05, *p* <.001. The sign of the coefficient suggests that, while older adults still had the highest levels of state curiosity, middle-aged adults had lower state curiosity than younger adults (see Fig 1B – solid line).

## Discussion

In the current study, we found a positive relationship between trait curiosity and state curiosity, indicating overlap of these constructs. Nevertheless, we found that the two types of curiosity are related to age in the opposite direction. Specifically, the results supported our preregistered hypothesis that state curiosity triggered by trivia questions is positively associated with age, while trait curiosity is negatively associated with age. We also found a quadratic relationship between age and state curiosity, such that older adults exhibit much higher state curiosity than middle aged adults, while middle aged adults may have the lowest levels, though it is important to note that this analysis was exploratory and should be replicated in future work. The overall positive relationship between state and trait curiosity here is important to demonstrate for a couple of reasons. First, it gives some additional validity to our measure of state curiosity that there is some overlap between those who are more interested in learning trivia and those who are more curious overall. Second, cohort effects and age differences in response bias are possible in a cross-sectional study. However, the fact that we find overlap of trait and state curiosity here in the same individuals supports the idea that response bias cannot fully account for our findings.

These findings indicate a nuanced relationship between aging and curiosity. Specifically, age does not have a uniform influence on curiosity; rather, we need to consider the multifaceted nature of this construct when discussing aging effects. In fact, there has been a growing number of studies indicating that developmental trajectories of curiosity, or information-seeking behavior, strongly depends on the type of curiosity researchers investigate [9,64]. This is because curiosity subsumes different levels of psychological processes (e.g., emotional processes, reinforcement, learning, attention, appraisal, etc.), each of which would be impacted differently by age [41]. For example, using an experimental information search task, a previous study showed that, while younger adults become curious about learning completely new topics, older adults are more motivated to deepen their existing knowledge [47]. It should be noted that our exploratory results also revealed a nonlinear relationship between age and state curiosity, showing a slight dip in state curiosity in middle age, followed by a more dramatic increase into older ages, while we did not find any such relationship in trait curiosity. This further highlights the nature of state curiosity as distinct from trait curiosity and suggests that state curiosity may be influenced by other factors. For example, middle aged adults may be more stressed and less happy, which may contribute to their experience of momentary curiosity [65,66], though future research is needed to further investigate the reasons for the nonlinear trend. Taken together, the evidence observed here further supports that trait and state curiosity may be reflective of different psychological processes, which may have different patterns of change across the lifespan.

One limitation of our findings is that we assessed state curiosity only in the context of trivia questions. This was a deliberate choice, as our hypothesis is based on the previous findings that people tend to be more curious about information for which they have more prior knowledge [11,38–40]. With the use of a wide range of trivia questions, participants likely had some prior knowledge about the general domains of these questions, although not of the exact questions themselves, as we removed items for which participants already knew the answer. However, future studies should examine which types of state curiosity are positively associated with age. For example, a previous study reported an age-related increase in curiosity toward other materials besides trivia questions [e.g., magic tricks; 67]. A related point is that we did not explicitly test for learning, as our primary question was related to curiosity and not learning. However, previous studies have used the same questions and paradigm with different age groups and shown that both younger and older adults learn the answers to the trivia questions at similar rates, even when they are not aware there will be a later memory test [35,68]. We also instructed participants that their goal was to learn trivia, even though they were not actually tested. While we did not examine the exact mechanisms through which age is positively associated with state curiosity in the current study, we conjectured that increased prior knowledge for the learning materials not only increased the motivation to learn the information, but also reduced the cost to engage in the information, but further research is needed to investigate that possibility.

Additionally, we did not screen participants for age-related or other cognitive impairment. However, we make the assumption here that participants, especially older adults, who were capable of finding and successfully completing a lengthy computer task were likely not impaired. This assumption is also supported by some work showing that older adults from online samples perform similarly to those in a laboratory setting on cognitive assessments [69], though samples can vary in terms of their effort in tasks administered online [see 70]. We also included multiple data checks to ensure data were good quality (which resulted in many exclusions). Still, it is possible that results may differ for individuals who do have a form of age-related cognitive decline, and future research may explore how measures of curiosity may change in learning-impaired conditions.

In sum, the current study finds different relationships between age and state and trait curiosity. Given that previous findings regarding the benefits of curiosity in older age [see 14] have generally been based on trait curiosity, it is important to consider some of the ways that older adults may maintain curiosity other than via trait curiosity measures. Specifically, older adults may be selectively curious about things that have greater self-relevance [33,42] or which are relevant to their prior knowledge [47], which may benefit them in specific contexts. For example, if an older adult is curious about gardening, they may be more likely to read gardening magazines, join a gardening group, or to learn a new gardening skill. Engaging with any of these activities can be beneficial for overall well-being and cognitive outcomes in older age, for example by fostering social connections [71,72] and learning complex new skills [17]. Thus, it may be important to recognize and encourage specific domains of curiosity for older adults, rather than simply focusing on measures of trait curiosity.
